# Psychological distress, tobacco smoking and alcohol use: A population survey in Great Britain

**DOI:** 10.1016/j.abrep.2025.100604

**Published:** 2025-04-05

**Authors:** Erikas Simonavičius, Parvati R. Perman-Howe, Deborah Robson, Ann McNeill, Loren Kock, Jamie Brown, Leonie S. Brose

**Affiliations:** a King‘s College London, Addiction Sciences Building, 4 Windsor Walk, Denmark Hill Campus, London SE5 8AF, United Kingdom; b University of Sheffield, School of Medicine and Population Health, Regent Court, 30 Regent Street, Sheffield S1 4DA, United Kingdom; c University College London, 1-19 Torrington Place, WC1E 7HB, United Kingdom

**Keywords:** Alcohol, AUDIT, Mental health, Psychological distress, Smoking, Tobacco

## Abstract

**Aims:**

Psychological distress, smoking and alcohol use are interconnected. This study explores how distress and smoking independently, and in combination, are associated with alcohol consumption, past attempts and motivation to restrict alcohol use among people in Great Britain.

**Methods:**

Pooled cross-sectional data of people (aged ≥ 16) in Great Britain (N = 87326) collected monthly from April 2020 to June 2023 in the nationally representative Smoking and Alcohol Toolkit Study. Multinomial and binary logistic regressions assessed how past 30-day distress (Kessler Psychological Distress Scale) and smoking were associated with alcohol use risk level (AUDIT-C), past-year attempts to restrict alcohol use, and motivation to restrict alcohol use in three months, adjusting for sex, age, socioeconomic and geographic characteristics.

Findings.

The interaction between distress and smoking on alcohol use risk level was significant. At all distress levels, odds of using alcohol at increasing or high risk were higher among participants who smoked in the past or currently. Moderate or serious distress was associated with lower odds of using alcohol at increasing risk among participants who did not smoke. Higher distress was positively associated with past-year attempts to restrict alcohol use, while smoking in those reporting low–to–moderate distress was negatively associated with past-year attempts. Higher distress was positively associated with motivation to restrict alcohol use in three months.

**Conclusions:**

Smoking currently or in the past was independently associated with increased odds of using alcohol at increasing- or high-risk levels. Among those not smoking, higher distress was associated with reduced odds of using alcohol at increasing risk.

## Highlights

• Smoking in the past or now increases odds of using alcohol at higher risk levels.
• Distress increases odds of using alcohol at higher risk, notably among smokers.
• Smokers with lower/moderate distress less often attempt to restrict alcohol use.
• Stronger distress is positively associated with attempts to restrict alcohol use.
• Stronger distress is positively associated with motivation to restrict alcohol use.

## 1 Introduction

Globally, alcohol use is the biggest risk factor for ill-health, disability and early mortality among 25- to 49-year-olds, and the ninth biggest risk factor for ill-health across all ages (Murray et al., 2020). According to the World Health Organisation, no safe alcohol consumption levels exist, and any use of alcohol affects health and cancer risk (Anderson et al., 2023). The Chief Medical Officer in the UK advise not to drink more than 14 units of alcohol a week on a regular basis, which is considered the ‘low risk’ alcohol use level (Department of Health, 2016). Alcohol misuse, including drinking at harmful and dependent levels, is a risk factor for mental health conditions, including depression, anxiety and psychoses, and is associated with increased risk of suicide (Puddephatt et al., 2022, Rizk et al., 2021). Conversely, mental ill-health is a risk factor for alcohol misuse, in part due to the belief that alcohol can mitigate symptoms of mental ill-health (Puddephatt et al., 2022). Available evidence suggests a bi-directional relationship between alcohol misuse and mental ill-health, with stronger indication that poor mental health increases alcohol use (Bell & Britton, 2014). In the United Kingdom, the relationship between alcohol use and mental health is borne out in the high prevalence of co-existing alcohol use disorders and mental ill-health (Puddephatt et al., 2021); for example, 71 % of people who started treatment for an alcohol use disorder in England in 2022/2023 also needed treatment for mental ill-health (Office for Health Improvement & Disparities, 2023). Similar relationship exists at the subclinical mental health level, where alcohol use is often perceived as a tool to cope with stress (Algren et al., 2018).

Tobacco smoking is another health-risk behaviour which is commonly associated with both mental ill-health (Richardson et al., 2019, College, 2013, Taylor et al., 2014) and alcohol consumption (Beard et al., 2017, Falk et al., 2006). Despite misperceptions that smoking helps regulating low mood or anxiety (which are symptoms of nicotine withdrawal), evidence strongly indicates that stopping smoking is associated with improvements in mental health (Taylor et al., 2014), and continued smoking is implicated in increased risk of poor mental health later in life (Lee et al., 2022). People who smoke are also more likely to consume alcohol, consume it daily and at greater quantities, and are at higher risk of alcohol misuse or dependence (McKee & Weinberger, 2013). A recent study of a nationally representative sample in England showed substantially elevated rates of psychological distress and diagnosed mental health conditions among the 4.6 % of adults who smoke and use alcohol at increasing risk compared with all adults (Garnett et al., 2024). Given the potential health risks of psychological distress, tobacco smoking and alcohol misuse separately, it is important to clarify the interplay between these factors and how they affect health in combination. Using most recent data from the same Great Britain (GB) population survey, this study aims to assess the associations between past 30-day psychological distress and smoking status with 1) alcohol consumption risk level (Research Question 1); 2) attempts to restrict alcohol consumptions in the past 12 months (RQ2); 3) motivation to restrict alcohol consumption in the next 3 months (RQ3).

## 2 Methods

### 2.1 Study design

This is a cross-sectional study using pooled data from 39 waves collected monthly from April 2020 to June 2023 in repeated household surveys from the Smoking and Alcohol Toolkit Study (STS/ATS). The STS/ATS is a nationally representative telephone survey of people aged 18 and older in GB (16 and older since January 2022), each month surveying around 1700 participants in England, 450 in Scotland and 300 in Wales on smoking and alcohol use behaviours. The study uses a hybrid of random probability and simple quota sampling, and data have been collected via computer assisted telephone interviews. Detailed survey protocol including methods and survey questions are described elsewhere (Beard et al., 2015, Kock et al., 2021).

Ethical approval for the STS/ATS was granted by the UCL Ethics Committee (ID 0498/001). The inclusion of the mental health module was approved by the same committee (2808/005).

### 2.2 Measures

#### 2.2.1 Covariates

*Sex:* participants’ sex was categorised into 1) female; 2) male; 3) other.

*Age:* measured as a continuous variable and recoded into a categorical variable with the following age groups: 1) 16–24; 2) 25–34; 3) 35–44; 4) 45–54; 5) 55–64; 6) ≥ 65. The sample included 632 participants aged 16 or 17.

*Ethnicity:* measured as a categorical variable with a list of ethnic groups used in the latest UK census (Government of the UK, 2023) and recoded into the following ethnicity groups: 1) White; 2) Asian/Asian British; 3) Black/Black British/Caribbean or African; 4) Multiple ethnic groups; 5) Other ethnic groups; 6) Don’t know, refused or unknown. Participants whose ethnicity is unknown (*n* = 8459) are from April to August waves in 2020, when ethnicity was not measured.

*Socioeconomic status: m*easured by categories using the National Readership Survey social grade system (National Readership Survey, 2016): A: higher managerial, administrative or professional; B: intermediate managerial, administrative or professional; C1: supervisory or clerical and junior managerial administrative or professional; C2: skilled manual workers; D: semi and unskilled manual workers; and E: casual or lowest grade workers, pensioners and others who depend on the welfare state for their income. For analyses, recoded into 1) ABC1 (professional to clerical occupations); 2) C2DE (manual occupations).

*Geographic region:* measured using categories of government office regions with the addition of Scotland and Wales. For analyses, recoded into 1) North England (North East, North West, Yorkshire and The Humber); 2) Central England (East Midlands, West Midlands, East of England); 3) South England (South East, South West); 4) London; 5) Scotland; 6) Wales.

#### 2.2.2 Primary predictors

Full descriptions of questions asked, and original response options are provided in the Supplement.

*Past 30-day distress:* measured using the Kessler Psychological Distress screening scale (K6)(Kessler et al., 2002, Kessler et al., 2003). A total score on the K6 from 0 to 24 was recoded as 1) low psychological distress (0 to 4); 2) moderate psychological distress (5 to 12); 3) serious psychological distress (13 to 24) (Brose et al., 2020, Perman-Howe et al., 2022, Prochaska et al., 2012).

Among participants from Scotland and Wales, past 30-day distress was measured from October 2020 therefore the first six waves used in the study do not include participants from those countries.

*Smoking status:* was defined as 1) currently smoking (smoking non-daily or daily tobacco cigarettes, pipe, cigar or shisha); 2) smoked in the past; 3) not smoking (never smoked).

#### 2.2.3 Outcome variables

*Alcohol consumption risk level:* measured using the first three questions from the Alcohol Use Disorders Identification Test (AUDIT), which is known as Alcohol Use Disorders Identification Test Consumption (AUDIT-C) (Bush et al., 1998)*.* The AUDIT-C has been shown to have excellent discriminative validity (Bradley et al., 2007) and excellent test–retest reliability (Simon et al., 2024). The combined score from 0 to 12 was recoded as 1) low risk (0 to 4); 2) increasing risk (5 to 7); high risk (8 to 12) levels (Bradley et al., 2007). This variable was also used as a covariate addressing RQ2 and RQ3.

*Attempts to restrict alcohol consumption:* participants who reported any drinking (i.e. at least ‘monthly or less’) were asked “How many attempts to restrict your alcohol consumption have you made in the last 12 months e.g., by drinking less, choosing lower strength alcohol or using smaller glasses?” Responses were coded as 1) no past-year attempts (responses 0 and “don’t know”); 2) at least one past-year attempt (responses 1 or more); participants who refused to answer were excluded from analyses. This variable was also used as a covariate addressing RQ3.

*Motivation to restrict alcohol consumption:* participants who reported any drinking (i.e. at least ‘monthly or less’) were asked about their motivation to restrict alcohol consumption using the adapted Motivation to Stop Scale (Kotz et al., 2013). Responses were coded as: 1) motivated to restrict alcohol use in the next 3 months; 2) not motivated to restrict alcohol use in the next 3 months; participants who did not know or refused to answer were excluded from analyses.

### 2.3 Statistical analysis

Analyses were preformed using R version 4.3.0. The STS/ATS uses marginal weighting with iterative adjustments to collect the sample that match the population of Great Britain in terms of key demographics (Fidler et al., 2011). Study sample (*N* = 87326) was described using weighted proportions and unweighted counts. All variables (except psychological distress, which was not collected from participants in Scotland and Wales for the first 6 waves of this period) had <5 % of missing cases that were excluded from complete case analyses. The sample flow diagram (Fig. 1, Supplements) outlines subsample sizes, and the following methods were used to address the research questions:

*RQ 1* included participants who answered at least one of the three AUDIT-C questions (*n* = 84401). To explore how past 30-day distress (reference category: ‘low’) and smoking (ref.: ‘not smoking’) independently and in interaction are associated with the risk level of alcohol consumption (ref.: ‘low risk’), a multinomial logistic regression was conducted using complete-case analysis, excluding cases with missing covariate data. Only statistically significant interactions between distress and smoking were retained in the model (Hosmer Jr et al., 2013).

*RQs 2 & 3* included the subsample of participants who reported having a drink at least ‘monthly or less’ (*n =* 30352)*.* To explore how past 30-day distress (ref.: ‘low’) and smoking (ref.: ‘not smoking’) independently and in interaction are associated with attempts to restrict alcohol use in the past 12 months (ref.: no attempts; *RQ2*) or being motivated to restrict alcohol use in the next 3 months (ref.: not motivated to restrict alcohol use in the next 3 months; *RQ3*), two multivariable logistic regressions were conducted using complete case analyses. The model addressing *RQ2* also adjusted for alcohol consumption risk level, and the model addressing *RQ3* adjusted for alcohol consumption risk level and past attempts to restrict alcohol use. Statistically significant interactions between distress and smoking were retained in the models (Hosmer Jr et al., 2013).

Additional analysis of most common reasons for past-year attempts to restrict alcohol use is provided in the Supplement Table 3.

## 3 Results

### 3.1 Sample characteristics

Table 1 summarises sociodemographic, psychological distress, smoking and alcohol use characteristics of the overall study sample (*N* = 87326). Weighted responses to the K6 psychological distress scale showed that 69.2% of participants reported low, 24.2% moderate and 6.7% serious distress in the past 30 days. Around 57.7% of participants reported never smoking, 25.1% smoked in the past and 16.4% were currently smoking. Around two-thirds of participants (67.4%) were in the no or low risk category of alcohol consumption, including 23.9% (*n* = 20032) of the sample who reported never having a drink containing alcohol, 21.7% were using alcohol at an increasing risk level and 10.9% at a high-risk level (Table 1). Most participants who answered questions about attempts and motivation to restrict alcohol consumption had not tried to restrict consumption in the past 12 months (70.6%) or did not want to cut down (66.4%).

**Table 1:** Participants' characteristics (*N* = 87326).

**Characteristic**	**Weighted % (unweighted *n*)**
**Sex**	
Male	48.62 (41948)
Female	50.73 (44634)
Other	0.65 (568)
Missing*	0.20^#^ (176)
**Age group**	
16–24	12.11 (8909)
25–34	16.94 (12151)
35–44	15.78 (11880)
45–54	16.74 (15133)
55–64	15.31 (15185)
≥65	23.12 (23927)
Missing*	0.15^#^ (141)
**Region**	
Central England	26.60 (19562)
South England	23.34 (17313)
North England	24.23 (17916)
London	13.81 (10473)
Scotland	7.72 (14283)
Wales	4.31 (7779)
**Ethnicity**	
White	77.29 (69057)
Asian/Asian British	4.63 (3381)
Black/Black British/Caribbean or African	3.93 (2955)
Multiple ethnic groups	2.32 (1794)
Other ethnic groups	1.20 (914)
Don’t know/Refused/Unknown^a^	10.63 (9225)
**Socioeconomic status**	
AB	27.52 (20994)
C1	26.45 (31078)
C2	21.31 (13939)
D	15.08 (7238)
E	9.64 (8919)
Missing*	4.44^#^ (5158)
**Past 30-day psychological distress**	
Low	69.16 (53325)
Moderate	24.18 (17193)
Serious	6.67 (4516)
Don’t know/Refused/Missing^b^*	7.98^#^ (12292)
**Smoking status**	
Not smoking	57.73 (50858)
Smoked in the past	25.08 (22716)
Currently smoking	16.35 (13059)
Don’t know*	0.84 (693)
**AUDIT-C category**	
No/Low risk (0–4 score)	67.44 (56665)
Increasing risk (5–7 score)	21.68 (18534)
High risk (8–12 score)	10.88 (9202)
Missing*	3.38^#^ (2925)
**Attempts to restrict alcohol consumption in the past 12 months**	
0	70.55 (21653)
1	11.03 (3223)
2	4.84 (1391)
3	2.46 (726)
≥4	5.23 (1568)
Don’t know	5.90 (1791)
Not asked this question*	66.61^#^ (56974)
**Motivation to restrict alcohol consumption**	
I REALLY want to cut down on drinking alcohol and intend to in the next month	7.19 (2113)
I REALLY want to cut down on drinking alcohol and intend to in the next 3 months	2.76 (818)
I want to cut down on drinking alcohol and hope to soon	2.91 (865)
I REALLY want to cut down on drinking alcohol but I don't know when I will	2.02 (586)
I want to cut down on drinking alcohol but haven't thought about when	3.85 (1122)
I think I should cut down on drinking alcohol but don't really want to	8.87 (2662)
I don't want to cut down on drinking alcohol	66.38 (20312)
Don't know*	4.68 (1435)
Refused*	1.33 (439)
Not asked this question*	66.61^#^ (56974)

Notes: * denotes categories that were excluded from data analyses. ^#^ missing cases were not included in overall frequencies, but weighted proportions of missing cases for each variable were provided.

a Participants’ ethnicity was not collected from April to August in 2020 but was included in analyses as “Don’t know/Refused/Unknown” category.

b Past 30-day distress was not measured among participants from Scotland and Wales in the first 6 waves, and they were categorised as ‘Missing’ together with those who responded “Don’t know” or “Prefer not to say”.

### 3.2 RQ1: Association of past 30-day distress and smoking status with alcohol consumption risk level

An interaction between past 30-day distress and smoking status was statistically significant (*χ^2^*(8) = 49.8, p < 0.001). Within all levels of past 30-day distress, smoking in the past or current smoking was associated with higher odds of consuming alcohol at increasing- or high-risk levels compared with participants who were not smoking (Table 2, Supplement Fig. S2). Compared with participants who were not smoking, the highest odds of increasing (aOR_current_ = 1.85, 95% CI: 1.52–2.25) and high risk alcohol use (aOR_current_ = 3.82, 95% CI: 3.01–4.86) were found in participants who currently smoked and experienced moderate distress (Table 2).

**Table 2:** Interaction between past 30-day distress and smoking status on the “Increasing” and “High risk” AUDIT-C scores (reference category: “Low risk”) in a multinomial regression model (*n* = 68278).

**Characteristic**	**Alcohol consumption risk level**				
	**Low (reference category)**	**Increasing**		**High risk**	
	**% (*n*)**	**% (*n*)**	**aOR (95% CI)**	**% (*n*)**	**aOR (95% CI)**
**Interactions within distress categories by smoking status**					
**Intercept**			**0.028 (0.025**–**0.031)**		**0.005 (0.004**–**0.006)**
**Low distress**					
Not smoking	70.7 (21077)	21.3 (6337)	Ref	8.0 (2398)	Ref
Smoked in the past	59.6 (7900)	26.3 (3485)	**1.61 (1.53**–**1.69)**	14.1 (1866)	**2.27 (2.12**–**2.43)**
Currently smoking	57.3 (3232)	24.2 (1364)	**1.37 (1.28**–**1.47)**	18.6 (1049)	**2.75 (2.53**–**3.00)**
*Total*	*66.1 (32209)*	*23.0 (11186)*		*10.9 (5313)*	
**Moderate distress**					
Not smoking	73.3 (6296)	19.6 (1686)	Ref	7.1 (611)	Ref
Smoked in the past	63.1 (2488)	24.5 (968)	**1.55 (1.32**–**1.81)**	12.4 (487)	**2.13 (1.72**–**2.65)**
Currently smoking	53.1 (1641)	26.6 (824)	**1.85 (1.52**–**2.25)**	20.3 (628)	**3.82 (3.01**–**4.86)**
*Total*	*66.7 (10425)*	*22.3 (3478)*		*11.0 (1726)*	
**Serious distress**					
Not smoking	76.8 (1281)	15.5 (258)	Ref	7.8 (130)	Ref
Smoked in the past	70.0 (649)	18.3 (170)	**1.37 (1.04**–**1.81)**	11.7 (108)	**1.73 (1.21**–**2.45)**
Currently smoking	59.3 (798)	20.5 (276)	**1.71 (1.31**–**2.26)**	20.1 (271)	**3.27 (2.35**–**4.59)**
*Total*	*69.2 (2728)*	*17.9 (704)*		*12.9 (509)*	
**Interactions within smoking categories by distress levels**					
**Not smoking**					
Low distress	70.7 (21077)	21.3 (637)	Ref	8.0 (2398)	Ref
Moderate distress	73.3 (6296)	19.6 (1686)	**0.86 (0.81**–**0.92)**	7.1 (611)	**0.88 (0.80**–**0.97)**
Serious distress	76.8 (1281)	15.5 (258)	**0.68 (0.59**–**0.79)**	7.8 (130)	0.96 (0.79–1.16)
*Total*	*71.5 (28654)*	*20.7 (8281)*		*7.8 (3139)*	
**Smoked in the past**					
Low distress	59.6 (7900)	26.3 (3485)	Ref	14.1 (1866)	Ref
Moderate distress	63.1 (2488)	24.5 (968)	**0.83 (0.70**–**0.98)**	12.4 (487)	0.83 (0.65–1.06)
Serious distress	70.0 (649)	18.3 (170)	**0.58 (0.40**–**0.85)**	11.7 (108)	0.73 (0.45–1.17)
*Total*	*60.9 (11037)*	*25.5 (4623)*		*13.6 (2461)*	
**Currently smoking**					
Low distress	57.3 (3232)	24.2 (1364)	Ref	18.6 (1049)	Ref
Moderate distress	53.1 (1641)	26.6 (824)	1.16 (0.96–1.41)	20.3 (628)	1.22 (0.95–1.57)
Serious distress	59.3 (798)	20.5 (276)	0.85 (0.60–1.22)	20.1 (271)	1.14 (0.73–1.77)
*Total*	*56.2 (5671)*	*24.4 (2464)*		*19.3 (1948)*	

Notes: the multinomial regression model was adjusted for sex, age, ethnicity, socioeconomic status and geographical region; the full model is provided in the Supplement. Adjusted odds ratios (aOR) in **bold** are statistically significant at *p* < 0.05.

Within smoking status categories, increasing levels of distress were associated with lower odds of consuming alcohol at increasing risk levels, but only among those who did not smoke or smoked in the past (Table 2). For example, among those who did not smoke, compared with participants reporting low past 30-day distress, increasing levels of distress were associated with lower odds of consuming alcohol at increasing-risk level (aOR_moderate_ = 0.86, 95% CI: 0.81–0.92; aOR_serious_ = 0.68, 95% CI: 0.59–0.79) but the associations were attenuated at high-risk alcohol use levels (aOR_moderate_ = 0.88, 95% CI: 0.80–0.97; aOR_serious_ = 0.96, 95% CI: 0.79–1.16).

### 3.3 RQ2: Association of past 30-day distress and smoking status with attempts to restrict alcohol use

An interaction between past 30-day distress and smoking status on past-year attempts to restrict alcohol use was statistically significant (*χ^2^*(4) = 26.7, p < 0.001). Compared with participants who did not smoke, current smoking was associated with lower odds of past-year attempts to restrict alcohol use in those who reported low (aOR_current_ = 0.50, 95% CI: 0.43–0.56) or moderate past 30-day distress (aOR_current_ = 0.62, 95% CI: 0.54–0.72). In those with serious past 30-day distress, attempts to restrict alcohol use in the past year were similar according to smoking status (Table 3, Supplement Fig. S3).

Within all smoking status categories, increasing past 30-day distress (compared with low distress level) was associated with higher odds of reporting attempts to restrict alcohol use in the past year (Table 3). The odds were highest among participants who were currently smoking: aOR = 2.04, 95% CI: 1.71–2.42 for moderate and aOR = 3.52, 95% CI: 2.82–4.40 for serious past 30-day distress compared with low distress.

**Table 3:** Interaction between past 30-day distress and smoking status on attempts to restrict alcohol use in the past 12 months (reference category: “No past-year attempts”) in a multivariable logistic regression model (*n* = 24145).

**Characteristic**	**Attempt(s) to restrict alcohol use in the past 12 months**	
	**% (*n*) attempted**	**aOR (95% CI)**
**Interactions within distress categories by smoking status**		
**Intercept**		**0.10 (0.08**–**0.12)**
**Low distress**		
Not smoking	21.6 (2053)	Ref
Smoked in the past	23.9 (1305)	1.08 (0.997–1.17)
Currently smoking	13.9 (333)	**0.50 (0.43**–**0.56)**
*Total*	*21.3 (3691)*	
**Moderate distress**		
Not smoking	30.1 (779)	Ref
Smoked in the past	34.1 (518)	1.11 (0.96–1.27)
Currently smoking	24.4 (344)	**0.62 (0.54**–**0.73)**
*Total*	*29.8 (1641)*	
**Serious distress**		
Not smoking	29.5 (130)	Ref
Smoked in the past	35.3 (106)	1.19 (0.86–1.64)
Currently smoking	33.6 (177)	1.04 (0.79–1.38)
*Total*	*32.6 (413)*	
**Interactions within smoking categories by distress levels**		
**Not smoking**		
Low distress	21.6 (2053)	Ref
Moderate distress	30.1 (779)	**1.62 (1.46**–**1.79)**
Serious distress	29.5 (130)	**1.67 (1.33**–**2.08)**
*Total*	*23.6 (2962)*	
**Smoked in the past**		
Low distress	23.9 (1305)	Ref
Moderate distress	34.1 (518)	**1.65 (1.45**–**1.88)**
Serious distress	35.3 (106)	**1.83 (1.42**–**2.36)**
*Total*	*26.5 (1929)*	
**Currently smoking**		
Low distress	13.9 (333)	Ref
Moderate distress	24.4 (344)	**2.04 (1.71**–**2.42)**
Serious distress	33.6 (177)	**3.52 (2.82**–**4.40)**
*Total*	*19.7 (854)*	

Notes: the logistic regression model was adjusted for sex, age, ethnicity, socioeconomic status, geographical region and AUDIT-C category. The full model is provided in the Supplement. Adjusted odds ratios (aOR) in **bold** are statistically significant at *p* < 0.05.

In addition to distress and smoking, past year attempts to restrict alcohol use were most strongly associated with increasing- (aOR = 2.16, 95% CI: 1.94–2.42) and high-risk (aOR = 4.14, 95% CI: 3.69–4.65) alcohol use (Supplement Table 2).

### 3.4 RQ3: Association of past 30-day distress and smoking status with motivation to restrict alcohol use

An interaction between past 30-day distress and smoking status on motivation to restrict alcohol use was not statistically significant (*χ^2^*(4) = 7.2, p = 0.128) and was not retained in the final model. Reporting an intention to restrict alcohol use in the next 3 months was statistically significantly associated with participants’ past 30-day distress but not with smoking status (Table 4). Compared with participants reporting low past 30-day distress, those who reported moderate (aOR = 1.40, 95% CI: 1.20–1.63) or high levels of distress (aOR = 1.52, 95% CI: 1.10–2.08) had significantly higher odds of being motivated to restrict alcohol use in the next 3 months. Motivation to restrict alcohol use in the next 3 months was most strongly associated with attempting to restrict alcohol use in the past 12 months (aOR = 9.16, 95% CI: 8.32–10.1; Supplement Table 2).

**Table 4:** Associations between past 30-day distress and smoking status with motivation to restrict alcohol consumption in the next 3 months in a multivariable logistic regression model (*n* = 22799).

**Characteristic**	**Motivation to restrict alcohol use in the next 3 months**	
	**% (*n*) motivated**	**aOR (95% CI)**
**Intercept**		**0.03 (0.02**–**0.04)**
**Past 30-day psychological distress**		
Low	9.1 (1495)	Ref
Moderate	14.1 (736)	**1.40 (1.20**–**1.63)**
Serious	17.5 (207)	**1.52 (1.10**–**2.08)**
**Smoking status**		
Not smoking	10.3 (1211)	Ref
Smoked in the past	11.8 (809)	1.03 (0.91–1.17)
Currently smoking	10.1 (418)	0.90 (0.74–1.08)

Notes: the logistic regression model was adjusted for sex, age, ethnicity, socioeconomic status, geographical region, AUDIT-C category and past year attempts to restrict alcohol use. The full model is provided in the Supplement. Adjusted odds ratios (aOR) in **bold** are statistically significant at *p* < 0.05.

## 4 Discussion

In a nationally representative sample of adults in Great Britain, odds of consuming alcohol at increasing or high-risk levels were higher among those who smoked in the past or currently at all distress levels, but the associations were stronger among those reporting moderate and serious distress. Attempts to restrict alcohol use in the past 12 months and motivation to restrict alcohol use in the next 3 months were both positively associated with increasing past 30-day distress, current smoking showed a negative association with past attempts to restrict alcohol use among those with low or moderate distress when compared with not smoking. Using alcohol at increasing- or high-risk was positively associated with past attempts to restrict alcohol use, and having tried to restrict alcohol use in the past was the strongest predictor of motivation to restrict alcohol use in the future.

### 4.1 Association of past 30-day distress and smoking status with alcohol consumption risk levels

The identified strong associations between past or current smoking with increasing and high alcohol use risk affirm the existing evidence on the positive relationship between the two behaviours (Garnett et al., 2024, Garnett et al., 2022). The association between increasing distress and lower odds of using alcohol at increasing risk among those who do not smoke or smoked in the past, however, requires further elaboration. The positive association between stress and alcohol use is commonly explained as a coping strategy—a way to reduce increasing negative affect by consuming alcohol (Baker et al., 2004). However, a recent systematic review of longitudinal studies (Wolkowicz et al., 2022) found inconsistent evidence for supporting the positive association between stress and alcohol use and, in the reverse predictive pathway, concluded that greater alcohol use is strongly associated with reduced distress, aligning with the stress response dampening hypothesis positing that alcohol use might reduce subjective stress (Sher, 1987). If increasing alcohol use leads to a reduction in self-reported distress in the short term, it could explain the study’s finding of significantly higher odds of using alcohol at increasing risk level among participants with low rather than moderate or high distress levels. Alternatively, the negative association between distress and increasing-risk alcohol use highlights the need for both researchers and practitioners to consider alternative motivations for alcohol consumption beyond stress relief alone. In contrast, the identified association might result from the higher odds of attempting to restrict alcohol use by the more distressed participants (as found when addressing *RQ* *2*), which would lead to lower alcohol use risk among those reporting higher distress. To test the latter explanation, a longitudinal study assessing the effectiveness of attempts to restrict alcohol use among participants with different levels of distress with particular focus on those reporting serious distress levels, would need to be conducted.

A unique finding from the current study is the significant interaction effect between distress and smoking status on alcohol use risk. Increasing psychological distress seems to be associated with lower odds of using alcohol at increasing risk among those who do not smoke or smoked in the past. However, compared with those who do not smoke, smoking in the past or current smoking were found to have a stronger, positive effect on the alcohol use risk at all distress levels. This resulted in the particularly heightened odds of using alcohol at high risk levels among those who currently smoke and experience moderate or serious distress. While the interaction merits further examination using longitudinal methods, this finding could inform prevention of risky alcohol consumption. For those who smoke, for example, the risk of increasing alcohol use could be mitigated by interventions that address psychological distress or, indirectly, by supporting smoking cessation.

### 4.2 Association of past 30-day distress and smoking status with attempts to restrict alcohol use

Attempts to restrict alcohol use in the past year were strongly associated with increasing or high risk alcohol use levels and moderate or serious distress. Current smoking was associated with significantly lower odds of past attempts to restrict alcohol use but only among participants reporting low or moderate past 30-day distress levels. Smoking is strongly associated with alcohol co-use, and results of this study suggest that smoking might also be a barrier for attempting to restrict alcohol use in the general population and likely in clinical settings too. This is supported by the evidence linking smoking, alcohol use and attempts to restrict one of the two behaviours. For example, literature shows that alcohol use reduces chances of quitting smoking successfully (van Amsterdam & van den Brink, 2023), that smoking is known to predict poorer outcome in alcohol use disorder treatment (van Amsterdam & van den Brink, 2022) and that smoking cessation attempts are associated with decreases in alcohol consumption and alcohol-related problems (Vinci et al., 2024). Nevertheless, the interaction effect demonstrates that once participants who smoke pass the threshold for serious distress, their past-year attempts to restrict alcohol use no longer differ from participants who do not smoke or smoked in the past—around one out of three participants within the serious distress group reported past-year attempts to restrict alcohol use.

### 4.3 Association of past 30-day distress and smoking status with motivation to restrict alcohol use

Motivation to restrict alcohol use in the next 3 months was associated with past-year attempts to restrict alcohol use, increasing or high-risk alcohol use and increasing distress levels. These findings confirm results from longitudinal analysis showing that motivation to restrict alcohol use generates further attempts to reduce consumption, particularly among increasing risk drinkers (De Vocht et al., 2018). In contrast to the negative association between smoking and past-year attempts to restrict alcohol use (*RQ2*), smoking status was not associated with motivation to restrict alcohol use in the next 3 months. In clinical practice, these findings show that patients’ distress in combination with past experience to restrict alcohol use could be leveraged to encourage another motivated and better supported attempt to restrict alcohol use. Although motivation and attempts to restrict alcohol use tend to be positively associated (De Vocht et al., 2018), full regression models predicting these outcomes show that these behaviours differ and are associated with different sets of predictors.

### 4.4 Strengths and limitations

The main strength of this study is the use of recent, nationally representative survey data which provide a comprehensive view on the relationship between self-reported psychological distress, tobacco smoking and alcohol use among general population in Great Britain. The reported interaction effects between distress and smoking on participants’ alcohol use patterns complement the existing evidence and highlight the importance of further exploration of bi-directional links between psychological wellbeing and concurrent smoking and alcohol use behaviours.

Limitations include the cross-sectional nature of the data which limits the exploration of temporal or causal associations between distress, smoking and alcohol use. Also, assessing self-reported psychological distress in the past month and alcohol use in the past week creates imbalance in the timing of these measures, which is important when explaining ‘the time-course of the subjective stress-alcohol relationship’ (Wolkowicz et al., 2022). The study findings reflect associations between psychological distress, smoking and alcohol use measured over longer timeframes and cannot be used to explain more transient variations or infer causal relationships between these factors. The measure of smoking status did not take into account frequency of smoking or use of alternative tobacco and nicotine products that could have independent effects on distress (Becker et al., 2021) or alcohol use (Frie et al., 2022). The study used the same dataset and shares the limitation mentioned by Garnett and colleagues (Garnett et al., 2024): namely, a possibility of selection bias due to the tendency of population surveys to exclude participants at the higher risk of alcohol use disorder (e.g., homeless and hospitalised people) or for participants to under-report their drinking frequency and quantity (Livingston & Callinan, 2015), which might have resulted in an underestimation of people who use alcohol at increasing- or high–risk levels or those who are currently smoking. Due to relatively small sample sizes, minority ethnic groups were grouped together, which limited the examination of possible differences between ethnicities and cultures, as well as the generalisability of the findings.

Despite these limitations, the analysis of the large population-level data highlights important interactions between psychological distress and smoking, and how these factors are related to alcohol use patterns. Future longitudinal studies should explore how attempts to stop and stopping smoking influence psychological distress and alcohol use behaviours.

## 5 Conclusions

While smoking was associated with increased odds of using alcohol at higher risk levels regardless of psychological distress, the association was greater among those experiencing moderate or serious compared with low distress. Attempts to restrict alcohol use in the past year were associated with higher psychological distress, except for people who smoked and reported low or moderate distress—they were less likely to report past-year attempts to restrict alcohol use. Longitudinal research is needed to explore the moderating effect of smoking on the association between psychological distress and alcohol use behaviours.

## CRediT authorship contribution statement

**Erikas Simonavičius:** Writing – review & editing, Writing – original draft, Project administration, Methodology, Investigation, Formal analysis, Data curation, Conceptualization. **Parvati R. Perman-Howe:** Writing – review & editing, Writing – original draft, Investigation, Data curation, Conceptualization. **Deborah Robson:** Writing – review & editing, Writing – original draft, Supervision, Project administration, Investigation, Funding acquisition, Data curation, Conceptualization. **Ann McNeill:** Writing – review & editing, Writing – original draft, Supervision, Project administration, Investigation, Funding acquisition, Data curation, Conceptualization. **Loren Kock:** Writing – review & editing, Writing – original draft, Methodology, Investigation, Funding acquisition, Data curation, Conceptualization. **Jamie Brown:** Writing – review & editing, Writing – original draft, Supervision, Project administration, Methodology, Investigation, Funding acquisition, Data curation, Conceptualization. **Leonie S. Brose:** Writing – review & editing, Writing – original draft, Supervision, Methodology, Investigation, Funding acquisition, Conceptualization.

## Funding

This work was supported by the UK Prevention Research Partnership (MR/S037519/1), which is funded by the British Heart Foundation, Cancer Research UK, Chief Scientist Office of the Scottish Government Health and Social Care Directorates, Engineering and Physical Sciences Research Council, Economic and Social Research Council, Health and Social Care Research and Development Division (Welsh Government), Medical Research Council, National Institute for Health Research, Natural Environment Research Council, Public Health Agency (Northern Ireland), The Health Foundation and Wellcome. Data collection for the Smoking and Alcohol Toolkit Studies is supported by Cancer Research UK (C1417/A22962).

## Declaration of competing interest

The authors declare that they have no known competing financial interests or personal relationships that could have appeared to influence the work reported in this paper.

## Data Availability

Data will be made available on request. Data are available from the corresponding author on a reasonable request.
