# Supplementary material for: Psychological distress, tobacco smoking and alcohol use: A population survey in Great Britain
Source: Addict Behav Rep. 2025 Apr 5;21:100604. doi: 10.1016/j.abrep.2025.100604 (PMC12008546; doi:10.1016/j.abrep.2025.100604)
Supplement: Supplementary Data 1 [file mmc1.docx]

Study supplement:
Psychological distress, tobacco smoking and alcohol use: a population survey in Great Britain

# Methods

Full descriptions of questions asked and original response options are described below.

## Primary predictors

*Smoking status:* participants were asked “Which of the following best applies to you? Please note we are referring to cigarettes and other kinds of tobacco that you set light to and NOT electronic or ‘heat-not-burn’ cigarettes’ with responses a) I smoke cigarettes (including hand-rolled) every day; b) I smoke cigarettes (including hand-rolled), but not every day; c) I do not smoke cigarettes at all, but I do smoke tobacco of some kind (e.g. pipe, cigar or shisha); d) I have stopped smoking completely in the last year; e) I stopped smoking completely more than a year ago; f) I have never been a smoker (i.e. smoked for a year or more). For analyses, original responses were recoded into 1) currently smoking (responses a, b or c); 2) stopped in the past year (d); 3) not smoking (e or f).

*Past 30-day distress:* measured using the K6 screening scale of psychological distress in the past 30 days (16, 17). The scale included questions asking about “During the past 30 days, about how often, if at all, did you feel: nervous; hopeless; restless or fidgety; so depressed that nothing could cheer you up; that everything was an effort; worthless?”. For each statement participants chose one of the following: all of the time (scored 4); most of the time (scored 3); some of the time (scored 2); a little of the time (scored 1); none of the time (scored 0); don’t know or prefer not to say (excluded from analyses). The statements and response options were randomly presented with responses displayed in a forward or reverse order. A total score was obtained ranging from 0 to 24; scores between 0-4 were coded as 1) low psychological distress; scores 5 to 12 were coded as 2) moderate psychological distress; and scores 13 to 24 were coded as 3) serious psychological distress (18-20).
Past 30-day distress was measured among participants from Scotland and Wales from October 2020, therefore the first six waves used in the study do not include Scottish or Welsh participants.

## Outcome variables

*Alcohol consumption risk level:* measured using three alcohol consumption questions from the Alcohol Use Disorders Identification Test (AUDIT-C) (21)*.* The three questions, responses and corresponding scoring were:

1. “How often do you have a drink containing alcohol?”
   1. Never (scored 0)
   2. Monthly or less (1)
   3. 2 to 4 times a month (2)
   4. 2 to 3 times a week (3)
   5. 4 to 5 times a week (4)
   6. 6 or more times a week (4)
2. “How many standard drinks containing alcohol do you have on a typical day when you are drinking?”
   1. 1 to 2 (scored 0)
   2. 3 to 4 (1)
   3. 5 to 6 (2)
   4. 7 to 9 (3)
   5. 10 to 12 (4)
   6. 13 to 15 (4)
   7. 16 or more (4)
3. How often do you have six or more standard drinks on one occasion?
   1. Never (scored 0)
   2. Less than monthly (1)
   3. Monthly (2)
   4. Weekly (3)
   5. Daily or almost daily (4).

For analyses, the combined scores of participants were coded to 1) low risk (scored 0 to 4); 2) increasing risk (scored 5 to 7); high risk (scored 8 to 12) levels (22). This variable was also used as a covariate addressing RQ2 and RQ3.

*Attempts to restrict alcohol consumption in the past 12 months:* participants who reported having a drink at least ‘monthly or less’ were asked “How many attempts to restrict your alcohol consumption have you made in the last 12 months e.g., by drinking less, choosing lower strength alcohol or using smaller glasses? Please include all attempts you have made in the last 12 months, whether or not they were successful, AND any attempt that you are currently making”. Participants could respond from 0 to 99; “don’t know” or refuse to answer. For analyses, responses were coded as 1) no attempts to restrict alcohol consumption in the past year (included those responding “don’t know”); 2) at least one past-year attempt to restrict alcohol consumption (all responding 1 or more attempts in the past 12 months); participants who refused to answer were excluded from analyses. This variable was also used as a covariate addressing RQ3.

*Motivation to restrict alcohol consumption:* participants who reported having a drink at least ‘monthly or less’ were asked “Which of the following best describes you?” with responses a) I REALLY want to cut down on drinking alcohol and intend to in the next month; b) I REALLY want to cut down on drinking alcohol and intend to in the next 3 months; c) I want to cut down on drinking alcohol and hope to soon; d) I REALLY want to cut down on drinking alcohol but I don't know when I will; e) I want to cut down on drinking alcohol but haven't thought about when; f) I think I should cut down on drinking alcohol but don't really want to; g) I don't want to cut down on drinking alcohol; h) Don’t know; i) Refuse to answer. Responses were collapsed into: 1) motivated to restrict alcohol use in the next 3 months (responses a or b); 2) not motivated to restrict alcohol use in the next 3 months (responses c to g); participants who did not know or refused to answer were excluded from analyses.

*Reasons* *for restricting alcohol consumption:* participants who reported at least one past-year attempt to restrict alcohol consumption were asked “Which of the following, if any, do you think contributed to you making the most recent attempt to restrict your alcohol consumption, cut down or stop drinking altogether?” with multiple response options a) advice from a doctor\health worker; b) government TV\radio\press advert; c) a decision that drinking was too expensive; d) I knew someone else who was cutting down; e) health problems I had at the time; f) a concern about future health problems; g) something said by family\friends\children; h) a significant birthday or event; i) improve my fitness; j) help with weight loss; k) detox; l) to give up alcohol for a month (e.g. taking part in Dry January); m) the coronavirus outbreak; n) other (please specify).

## Study sample and missing data

A flow diagram describing sample selection for study research questions is provided in Figure 1.

Cases with missing data for attempts to restrict alcohol consumption (*n*=1,346)

Full study sample (*N*=87,326)

RQ1: participants who answered at least one AUDIT‑C question (*n*=68,278)

RQ2: participants who never have a drink containing alcohol (*n*=37,926)

RQ2: participants who reported drinking alcohol (*n*=24,145)

RQ3: participants who reported drinking alcohol (*n*=22,799)

Cases with missing covariates for complete case analysis *(n=*6,207*)*

RQ1: missing cases for AUDIT-C questions (*n*=2,925)

Cases with missing covariates for complete case analysis (*n*=16,123)

***Inclusion criteria and missing outcome data***

***Missing covariate data***

Figure 1 Sample flow diagram indicating participants’ flow when addressing study research questions (RQ).

## RQ1: association of past 30-day distress and smoking status with alcohol consumption risk level

Table 1 Full multinomial logistic regression model predicting “Increasing” and “High risk” AUDIT-C scores versus “Low risk” scores as a reference, adjusted for sociodemographic, smoking and psychological distress characteristics (n = 68278).

| **Characteristic** | **Low risk AUDIT-C (0-4; reference category)** | **Increasing risk AUDIT-C (5-7)** | | **High risk AUDIT-C (8-12)** | |
| --- | --- | --- | --- | --- | --- |
|  | **% (*n*)** | **% (*n*)** | **aOR (95% CI)** | **% (*n*)** | **aOR (95% CI)** |
| **Intercept** |  |  | **0.028 (0.025-0.031)** |  | **0.005 (0.004-0.006)** |
| **Past 30-day psychological distress** |  |  |  |  |  |
| Low | 66.1 (32209) | 23.0 (11186) | Ref | 10.9 (5313) | Ref |
| Moderate | 66.7 (10425) | 22.3 (3478) | **0.86 (0.81-0.92)** | 11.0 (1726) | **0.88 (0.80-0.97)** |
| Serious | 69.2 (2728) | 17.9 (704) | **0.68 (0.59-0.79)** | 12.9 (509) | 0.96 (0.79-1.16) |
| **Smoking status** |  |  |  |  |  |
| Not smoking | 71.5 (28654) | 20.7 (8281) | Ref | 7.8 (3139) | Ref |
| Smoked in the past | 60.9 (11037) | 25.5 (4623) | **1.61 (1.53-1.69)** | 13.6 (2461) | **2.27 (2.12-2.43)** |
| Currently smoking | 56.2 (5671) | 24.4 (2464) | **1.37 (1.28-1.47)** | 19.3 (1948) | **2.75 (2.53-3.00)** |
| **Gender** |  |  |  |  |  |
| Female | 74.8 (26199) | 18.7 (6554) | Ref | 6.4 (2254) | Ref |
| Male | 57.5 (18918) | 26.6 (8735) | **1.87 (1.80-1.95)** | 15.9 (5230) | **3.29 (3.12-3.48)** |
| Other | 63.1 (245) | 20.4 (79)* | **1.33 (1.02-1.73)** | 16.5 (64)* | **2.75 (2.05-3.69)** |
| **Age group** |  |  |  |  |  |
| 16-24 | 61.7 (4225) | 24.4 (1675) | **2.96 (2.74-3.19)** | 13.9 (952) | **3.86 (3.48-4.27)** |
| 25-34 | 64.0 (6228) | 25.6 (2491) | **2.38 (2.23-2.54)** | 10.4 (1013) | **2.20 (2.00-2.42)** |
| 35-44 | 63.4 (6083) | 25.4 (2436) | **2.19 (2.05-2.34)** | 11.2 (1070) | **2.26 (2.06-2.48)** |
| 45-54 | 60.4 (7377) | 25.2 (3082) | **2.14 (2.01-2.27)** | 14.3 (1747) | **2.91 (2.69-3.16)** |
| 55-64 | 64.1 (7645) | 23.1 (2755) | **1.82 (1.71-1.93)** | 12.7 (1519) | **2.41 (2.22-2.62)** |
| ≥65 | 76.8 (13804) | 16.3 (2929) | Ref | 6.9 (1247) | Ref |
| **Region** |  |  |  |  |  |
| Central England | 68.5 (11872) | 21.6 (3738) | Ref | 9.9 (1716) | Ref |
| South England | 67.6 (10374) | 22.2 (3399) | 1.01 (0.95-1.06) | 10.2 (1565) | 1.01 (0.94-1.09) |
| North England | 63.1 (10026) | 23.3 (3710) | **1.17 (1.11-1.24)** | 13.6 (2153) | **1.50 (1.40-1.61)** |
| London | 68.4 (6162) | 21.9 (1975) | **1.11 (1.04-1.18)** | 9.6 (866) | **1.11 (1.02-1.22)** |
| Scotland | 62.6 (4384) | 25.2 (1767) | **1.25 (1.16-1.34)** | 12.1 (848) | **1.33 (1.21-1.46)** |
| Wales | 68.3 (2544) | 20.9 (779) | 1.00 (0.91-1.10) | 10.7 (400) | 1.12 (0.99-1.26) |
| **Ethnicity** |  |  |  |  |  |
| White | 64.5 (34099) | 23.7 (12507) | Ref | 11.8 (6250) | **3.83 (3.41-4.30)** |
| Asian/Asian British | 88.2 (2390) | 8.3 (226) | **0.19 (0.17-0.22)** | 3.5 (94)* | **0.16 (0.13-0.20)** |
| Black/Black British/Caribbean or African | 85.3 (2009) | 10.9 (257) | **0.28 (0.24-0.32)** | 3.8 (90)* | **0.21 (0.17-0.27)** |
| Multiple ethnic groups | 69.5 (989) | 20.9 (297) | **0.64 (0.56-0.74)** | 9.7 (138)* | **0.58 (0.48-0.71)** |
| Other ethnic groups | 80.9 (554) | 13.1 (90)* | **0.34 (0.27-0.43)** | 6.0 (41)* | **0.29 (0.21-0.41)** |
| Don’t know/Refused/Unknown^a^ | 64.5 (5321) | 24.1 (1991) | 1.04 (0.98-1.11) | 11.3 (935) | 0.99 (0.91-1.07) |
| **Socioeconomic status** |  |  |  |  |  |
| ABC1 | 63.4 (27782) | 25.0 (10967) | **1.57 (1.51-1.64)** | 11.6 (5100) | **1.48 (1.40-1.56)** |
| C2DE | 72.0 (17580) | 18.0 (4401) | Ref | 10.0 (2448) | Ref |
| **Distress * Smoking status** |  |  |  |  | ***χ^2^*(8) = 49.8, p<0.001** |
| **Interactions within distress categories by smoking status** | | | | | |
| **Low distress** |  |  |  |  |  |
| Not smoking | 70.7 (21077) | 21.3 (6337) | Ref | 8.0 (2398) | Ref |
| Smoked in the past | 59.6 (7900) | 26.3 (3485) | **1.61 (1.53-1.69)** | 14.1 (1866) | **2.27 (2.12-2.43)** |
| Currently smoking | 57.3 (3232) | 24.2 (1364) | **1.37 (1.28-1.47)** | 18.6 (1049) | **2.75 (2.53-3.00)** |
| **Moderate distress** |  |  |  |  |  |
| Not smoking | 73.3 (6296) | 19.6 (1686) | Ref | 7.1 (611) | Ref |
| Smoked in the past | 63.1 (2488) | 24.5 (968) | **1.55 (1.32-1.81)** | 12.4 (487) | **2.13 (1.72-2.65)** |
| Currently smoking | 53.1 (1641) | 26.6 (824) | **1.85 (1.52-2.25)** | 20.3 (628) | **3.82 (3.01-4.86)** |
| **Serious distress** |  |  |  |  |  |
| Not smoking | 76.8 (1281) | 15.5 (258) | Ref | 7.8 (130) | Ref |
| Smoked in the past | 70.0 (649) | 18.3 (170) | **1.37 (1.04-1.81)** | 11.7 (108) | **1.73 (1.21-2.45)** |
| Currently smoking | 59.3 (798) | 20.5 (276) | **1.71 (1.31-2.26)** | 20.1 (271) | **3.27 (2.35-4.59)** |
| **Interactions within smoking categories by distress level** | | | | | |
| **Not smoking** |  |  |  |  |  |
| Low distress | 70.7 (21077) | 21.3 (637) | Ref | 8.0 (2398) | Ref |
| Moderate distress | 73.3 (6296) | 19.6 (1686) | **0.86 (0.81-0.92)** | 7.1 (611) | **0.88 (0.80-0.97)** |
| Serious distress | 76.8 (1281) | 15.5 (258) | **0.68 (0.59-0.79)** | 7.8 (130) | 0.96 (0.79-1.16) |
| **Smoked in the past** |  |  |  |  |  |
| Low distress | 59.6 (7900) | 26.3 (3485) | Ref | 14.1 (1866) | Ref |
| Moderate distress | 63.1 (2488) | 24.5 (968) | **0.83 (0.70-0.98)** | 12.4 (487) | 0.83 (0.65-1.06) |
| Serious distress | 70.0 (649) | 18.3 (170) | **0.58 (0.40-0.85)** | 11.7 (108) | 0.73 (0.45-1.17) |
| **Currently smoking** |  |  |  |  |  |
| Low distress | 57.3 (3232) | 24.2 (1364) | Ref | 18.6 (1049) | Ref |
| Moderate distress | 53.1 (1641) | 26.6 (824) | 1.16 (0.96-1.41) | 20.3 (628) | 1.22 (0.95-1.57) |
| Serious distress | 59.3 (798) | 20.5 (276) | 0.85 (0.60-1.22) | 20.1 (271) | 1.14 (0.73-1.77) |

Note: adjusted odds ratios (aOR) in **bold** are statistically significant at *p*<0.05. Cells with asterisk (*) include low counts, and the respective odds ratios might not be reliable.

Figure 2 Interaction effects of smoking status within distress categories on the risk level of alcohol use.

## RQ2 & RQ3: association of past 30-day distress and smoking status with attempts to restrict alcohol use & association of past 30-day distress and smoking status with motivation to restrict alcohol use

*Table 2 Full multivariable logistic regression models predicting at least one attempt to restrict alcohol in the past 12 months compared with no attempts (n=24145) and motivation to restrict alcohol use in the next 3 months compared with no motivation to restrict alcohol use* (*n=22799*)*.*

| **Characteristic** | **% (n) attempted to restrict alcohol in the past 12 months** | **Multivariable associations**  **aOR (95% CI)** | **% (n) motivated to restrict alcohol use in the next 3 months** | **Multivariable associations**  **aOR (95% CI)** |
| --- | --- | --- | --- | --- |
| **Intercept** |  | **0.10 (0.08-0.12)** |  | **0.03 (0.02-0.04)** |
| **Past 30-day psychological distress** |  |  |  |  |
| Low | 21.3 (3691) | Ref | 9.1 (1495) | Ref |
| Moderate | 29.8 (1641) | **1.62 (1.46-1.79)** | 14.1 (736) | **1.40 (1.20-1.63)** |
| Serious | 32.6 (413) | **1.67 (1.33-2.08)** | 17.5 (207) | **1.52 (1.10-2.08)** |
| **Smoking status** |  |  |  |  |
| Not smoking | 23.6 (2962) | Ref | 10.3 (1211) | Ref |
| Smoked in the past | 26.5 (1929) | 1.08 (0.997-1.17) | 11.8 (809) | 1.03 (0.91-1.17) |
| Currently smoking | 19.7 (854) | **0.50 (0.43-0.56)** | 10.1 (418) | 0.90 (0.74-1.08) |
| **Gender** |  |  |  |  |
| Female | 25.3 (2532) | Ref | 12.1 (1136) | Ref |
| Male | 22.7 (3183) | **0.81 (0.76-0.86)** | 9.7 (1290) | **0.81 (0.74-0.89)** |
| Other | 21.6 (30)* | **0.63 (0.41-0.95)** | 9.4 (12)* | 0.66 (0.33-1.24) |
| **Age group** |  |  |  |  |
| 18-24 | 22.6 (594) | 1.05 (0.92-1.19) | 8.5 (214) | 0.85 (0.69-1.05) |
| 25-34 | 24.9 (899) | **1.36 (1.21-1.52)** | 10.6 (366) | 1.17 (0.97-1.40) |
| 35-44 | 28.1 (1022) | **1.67 (1.50-1.86)** | 13.3 (459) | **1.50 (1.27-1.78)** |
| 45-54 | 27.1 (1355) | **1.55 (1.40-1.72)** | 13.0 (611) | **1.54 (1.32-1.81)** |
| 55-64 | 23.7 (1061) | **1.38 (1.24-1.53)** | 11.8 (495) | **1.57 (1.34-1.85)** |
| ≥65 | 17.0 (814) | Ref | 6.5 (293) | Ref |
| **Region** |  |  |  |  |
| Central England | 23.9 (1348) | Ref | 10.4 (557) | Ref |
| South England | 25.4 (1298) | 1.09 (0.99-1.19) | 11.7 (562) | 1.11 (0.97-1.27) |
| North England | 24.0 (1391) | 0.93 (0.85-1.02) | 10.7 (590) | 1.01 (0.88-1.15) |
| London | 28.8 (846) | **1.20 (1.08-1.33)** | 12.5 (348) | 1.06 (0.90-1.24) |
| Scotland | 18.8 (595) | **0.72 (0.65-0.81)** | 8.7 (258) | 0.94 (0.79-1.11) |
| Wales | 17.9 (267) | **0.71 (0.61-0.83)** | 8.8 (123)* | 0.998 (0.80-1.24) |
| **Ethnicity** |  |  |  |  |
| White | 23.3 (4579) | Ref | 10.5 (1947) | Ref |
| Asian/Asian British | 25.2 (99)* | 1.12 (0.88-1.42) | 10.6 (39)* | 1.02 (0.70-1.46) |
| Black/Black British/Caribbean or African | 25.7 (123)* | **1.26 (1.00-1.56)** | 15.6 (68)* | **1.80 (1.32-2.42)** |
| Multiple ethnic groups | 27.2 (128)* | 1.15 (0.92-1.42) | 14.1 (62)* | 1.35 (0.99-1.81) |
| Other ethnic groups | 22.6 (35)* | 0.93 (0.62-1.36) | 9.3 (14)* | 0.87 (0.46-1.53) |
| Don’t know/Refused/Unknown | 26.2 (781) | 0.97 (0.88-1.06) | 10.9 (308) | 0.90 (0.78-1.04) |
| **Socioeconomic status** |  |  |  |  |
| ABC1 | 25.3 (4289) | **1.32 (1.23-1.42)** | 11.2 (1803) | 1.10 (0.99-1.22) |
| C2DE | 20.2 (1456) | Ref | 9.4 (635) | Ref |
| **AUDIT C category** |  |  |  |  |
| Low risk (1-4 score)* | 12.3 (457) | Ref | 6.1 (201) | Ref |
| Increasing risk (5-7 score) | 22.2 (3051) | **2.16 (1.94-2.42)** | 9.5 (1237) | **1.27 (1.07-1.50)** |
| High risk (8-12 score) | 33.3 (2237) | **4.14 (3.69-4.65)** | 15.4 (1000) | **1.77 (1.49-2.11)** |
| **Attempted to restrict alcohol in the past 12 months** |  |  |  |  |
| No attempts |  |  | 4.2 (733) | Ref |
| ≥1 attempts |  |  | 31.6 (1705) | **9.16 (8.32-10.1)** |
| **Distress * Smoking status** |  | ***χ^2^*(4) = 26.7, p<0.001** |  | *χ^2^*(4) = 7.2, p=0.128 |

Note: adjusted odds ratios (aOR) in **bold** are statistically significant at *p*<0.05. Cells with asterisk (*) include low counts, and the respective odds ratios might not be reliable.

Figure 3 Interaction effects of smoking status within distress categories on the past-year attempts to restrict alcohol use.

## Additional analysis: Common reasons for attempting to restrict alcohol use

Additionally, this study explored reasons for attempting to restrict alcohol consumption among participants who have made at least one attempt to restrict alcohol consumption in the past 12 months. This research question was not included in the manuscript due to small sample size and distant relevance to the three main research questions.

*Reasons for restricting alcohol consumption* were asked of participants who reported at least one past-year attempt to restrict alcohol consumption: “Which of the following, if any, do you think contributed to you making the most recent attempt to restrict your alcohol consumption, cut down or stop drinking altogether?” with multiple response options a) advice from a doctor\health worker; b) government TV\radio\press advert; c) a decision that drinking was too expensive; d) I knew someone else who was cutting down; e) health problems I had at the time; f) a concern about future health problems; g) something said by family\friends\children; h) a significant birthday or event; i) improve my fitness; j) help with weight loss; k) detox; l) to give up alcohol for a month (e.g. taking part in Dry January); m) the coronavirus outbreak; n) other (please specify).

The following analysis included participants who reported at least one past-year attempt to restrict alcohol consumption (*n=6899*), frequencies and counts (overall and by past 30-day distress levels using *χ^2^* test) were used to describe reasons for attempting to restrict alcohol use.

*Table 3 Frequency of reasons for attempting to restrict alcohol use in the last 12 months, overall and by reported psychological distress level (N=6899).*

| **Reason for attempting to restrict alcohol use in the past 12 months** | **% (n) overall** | **% (n) among different distress levels** | | | **Statistical test** |
| --- | --- | --- | --- | --- | --- |
|  |  | **Low distress** | **Moderate distress** | **Serious distress** |  |
| Help with weight loss | 43.2 (2981) | 45.8 (1775) | 41.6 (726) | 30.6 (137) | ***ꭕ^2^*(2)=40.8** |
| Improve my fitness | 43.1 (2973) | 44.4 (1721) | 43.7 (763) | 32.0 (143) | ***ꭕ^2^*(2)=25.2** |
| A concern about future health problems | 36.4 (2512) | 35.7 (1384) | 38.0 (664) | 38.7 (173) | *ꭕ^2^*(2)=3.8 |
| To give up alcohol for a month (e.g., taking part in Dry January) | 18.8 (1294) | 21.2 (823) | 16.2 (283) | 11.2 (50) | ***ꭕ^2^*(2)=39.0** |
| Detox | 17.1 (1180) | 16.1 (623) | 20.3 (354) | 17.7 (79) | ***ꭕ^2^*(2)=14.9** |
| A decision that drinking was too expensive | 14.2 (980) | 10.8 (419) | 18.3 (319) | 23.3 (104) | ***ꭕ^2^*(2)=91.8** |
| Health problems I had at the time | 13.1 (907) | 10.1 (391) | 14.9 (260) | 32.2 (144) | ***ꭕ^2^*(2)=179.5** |
| I knew someone else who was cutting down | 11.1 (765) | 10.0 (388) | 13.1 (229) | 11.9 (53) | ***ꭕ^2^*(2)=12.2** |
| Something said by family/friends/children | 10.0 (691) | 7.9 (305) | 12.3 (214) | 21.9 (98) | ***ꭕ^2^*(2)=98.5** |
| Other | 8.2 (567) | 7.2 (281) | 8.8 (153) | 12.3 (55) | ***ꭕ^2^*(2)=15.5** |
| Advice from a doctor or a health worker | 6.4 (439) | 5.2 (202) | 6.6 (116) | 15.0 (67) | ***ꭕ^2^*(2)=64.9** |
| No answer | 5.4 (370) | 5.5 (213) | 4.9 (86) | 4.7 (21) | *ꭕ^2^*(2)=1.1 |
| A significant birthday or event | 4.1 (286) | 3.7 (142) | 5.4 (94) | 6.9 (31) | ***ꭕ^2^*(2)=15.9** |
| Government TV/radio/press advert | 2.6 (181) | 2.6 (102) | 2.4 (44) | 2.5 (11) | *ꭕ^2^*(2)=0.09 |
| Own decision/nothing | 1.4 (97) | 1.4 (54) | 1.3 (22) | 2.0 (9) | *ꭕ^2^*(2)=1.5 |
| Don’t know | 0.5 (37) | 0.7 (26) | 0.3 (6) | 0.2 (1)* | *-* |
| Had a baby/pregnant | 0.4 (27) | 0.2 (9) | 0.8 (14) | 0.4 (2)* | *-* |
| Refused | 0.2 (14) | 0.2 (8) | 0.2 (3)* | 0.2 (1)* | *-* |
| Family problems | 0.1 (4) | 0* | 0.2 (4)* | 0* | - |

Note: statistical tests’ results in **bold** are statistically significant at *p*<0.05. * statistical comparisons were not made where participants’ counts for any of psychological distress groups were ≤5.

Concerns about health and fitness were the most mentioned reasons for attempting to restrict alcohol use in the past 12 months, with most participants choosing weight loss (43.2%), improving fitness (43.1%) and concerns about future health (36.4%, Table 3). Concerns about future health was equally important for participants with different psychological distress levels, while weight loss and fitness were more important for participants with low or moderate distress but less important for participants with serious distress. Some reasons for attempting to restrict alcohol use were more prominent for participants with serious rather than with low or moderate distress, including health problems they had at the time, something said by family, friends or children, advice from a doctor or health worker and a decision that drinking was too expensive (Table 3).

Participants who attempted to restrict alcohol use in the last year most commonly did so to lose weight, improve fitness or because they were concerned about future health. Reasons related to weight loss or fitness were most prominent among participants with low or medium distress, while participants reporting serious distress were significantly more motivated by current health problems, comments made by family or friends, advice from a doctor or health worker and because drinking was too expensive. Given the relevance of these reasons among most distressed participants, screening for alcohol use and alcohol-related concerns should be an important part of the assessment of people who attend mental health services because of their responsiveness to advice from medical professionals and willingness to address alcohol use. For people with low or medium distress, on the other hand, reasons related with physical health could be used as motivators to address alcohol use in general medical practice.
